# IL-33/ST2 signaling contributes to radicular pain by modulating MAPK and NF-κB activation and inflammatory mediator expression in the spinal cord in rat models of noncompressive lumber disk herniation

**DOI:** 10.1186/s12974-017-1021-4

**Published:** 2018-01-12

**Authors:** Si-Jian Huang, Jian-Qin Yan, Hui Luo, Lu-Yao Zhou, Jian-Gang Luo

**Affiliations:** 1 Department of Anesthesiology, The Second Xiangya Hospital, Central South University, Changsha, Hunan 410011 China; 2 Department of Anesthesiology, Xiangya Hospital, Central South University, Changsha, Hunan 410008 China

**Keywords:** IL-33/ST2 signaling, Radicular pain, MAPK, NF-κB, Inflammatory mediator, Lumber disk herniation

## Abstract

**Background:**

Immune and inflammatory responses occurring in the spinal cord play a pivotal role in the progression of radicular pain caused by intervertebral disk herniation. Interleukin-33 (IL-33) orchestrates inflammatory responses in a wide range of inflammatory and autoimmune disorders of the nervous system. Thus, the purpose of this study is to investigate the expression of IL-33 and its receptor ST2 in the dorsal spinal cord and to elucidate whether the inhibition of spinal IL-33 expression significantly attenuates pain-related behaviors in rat models of noncompressive lumbar disc herniation.

**Methods:**

Lentiviral vectors encoding short hairpin RNAs that target IL-33 (LV-shIL-33) were constructed for gene silencing. Rat models of noncompressive lumber disk herniation were established, and the spines of rats were injected with LV-shIL-33 (5 or 10 μl) on the first day after the operation. Mechanical thresholds were evaluated during an observation period of 21 days. Moreover, the expression levels of spinal tumor necrosis factor-α (TNF-α), interleukin-1β (IL-1β), interleukin-6 (IL-6), and cyclooxygenase 2 (COX-2) and the activation of the mitogen-activated protein kinases (MAPK) and nuclear factor-κB (NF-κB) pathways were evaluated to gain insight into the mechanisms related to the contribution of IL-33/ST2 signaling to radicular pain.

**Results:**

The application of nucleus pulposus (NP) to the dorsal root ganglion (DRG) induced an increase in IL-33 and ST2 expression in the spinal cord, mainly in the dorsal horn neurons, astrocytes, and oligodendrocytes. Spinally delivered LV-shIL-33 knocked down the expression of IL-33 and markedly attenuated mechanical allodynia. In addition, spinal administration of LV-shIL-33 reduced the overexpression of spinal IL-1β, TNF-α, and COX-2 and attenuated the activation of C-Jun N-terminal kinase (JNK), extracellular signal-regulated kinase (ERK), and NF-κB/p65 but not p38.

**Conclusions:**

This study indicates that spinal IL-33/ST2 signaling plays an important role in the development and progression of radicular pain in rat models of noncompressive lumber disk herniation. Thus, the inhibition of spinal IL-33 expression may provide a potential treatment to manage radicular pain caused by intervertebral disk herniation.

## Background

Radicular pain, most commonly induced by a herniated disk, is estimated to affect up to 2.2% of the general adult population, with substantial physical burdens and a heavy toll in terms of healthcare costs and work disability [1, 2]. Previous studies have shown that the mechanism of radicular pain is mostly due to the result of mechanical compression of the nerve roots by the herniated nucleus pulposus (NP) [3–5]. However, there is increasing evidence to support the idea that immune and inflammatory responses occurring in the peripheral and central nervous systems also play a pivotal role in the progress of radicular pain [6–8]. It has been demonstrated that interleukin-1β (IL-1β) [8–10], interleukin-6 (IL-6) [9], interleukin-8 (IL-8), [11], tumor necrosis factor-alpha (TNF-α) [10, 12, 13], phospholipase A2 [9], nitric oxide synthase (NOS) [9], and cyclooxygenase-2 (COX-2) [14] are activated in the dorsal root ganglion (DRG) and/or the spinal cord in response to lumbar disc herniation and that their altered expression is closely related to radicular pain.

Interleukin-33 (IL-33) is a new member of the IL-1 family of cytokines, which is demonstrated to be involved in the modulation of immune and inflammatory responses through its receptor complex that is composed of ST2 and the IL-1 receptor accessory protein (IL-1RAcp) [15, 16]. IL-33 expression is mediated through one or more of the mitogen-activated protein kinases (MAPK) (extracellular regulated kinase (ERK), p38 MAPK, c-Jun N-terminal kinase (JNK)) and nuclear factor-kappa B (NF-κB) [15, 17, 18]. IL-33/ST2 signaling plays a critical role in the development and progression of many inflammatory and autoimmune diseases of the nervous system, including experimental autoimmune encephalomyelitis, Alzheimer’s disease, and subarachnoid hemorrhage [19]. Notably, reports have indicated that IL-33/ST2 signaling is involved in the regulation of inflammatory pain in the peripheral and central nervous systems [20, 21]. Moreover, it is also shown that spinal IL-33/ST2 signaling plays a vital role in the modulation of neuropathic pain after spinal nerve ligation or chronic constriction injury of the sciatic nerve [22, 23]. However, to our knowledge, it is still unknown whether spinal IL-33/ST2 signaling also facilitates radicular pain that is caused by intervertebral disc herniation.

Thus, the aim of the present study was to test whether the inhibition of spinal IL-33 expression could significantly influence the development and progression of radicular pain, using a rat model of noncompressive lumbar disc herniation. We also evaluated the expression of IL-1β, IL-6, COX-2, and TNF-α and the activation of the MAPK and NF-κB pathways in the spinal cord of these rats to gain insight into the mechanisms related to the contributions of IL-33/ST2 signaling to radicular pain in this model system.

## Methods

### Lentiviral vector production

Lentiviral vectors were used to generate constructs encoding IL-33 small interfering RNAs (siRNAs) or control siRNA (scrambled sequence), as described previously [24]. Briefly, three independent siRNA sequences directed against the cDNA sequence of rat IL-33 (GenBank Accession NM_001014166.1) were designed using the Ambion application, following previously published guidelines [25]. The sequences of the three siRNAs are as follows: 5′-AATGTTGAAGATTGTGGGAAA-3′ (IL-33-siRNA1), 5′-ATGATGAGAGCTGTAACAATA-3′ (IL-33-siRNA2), and 5′-GTGAACATGAGTCCTATCAAA-3′ (IL-33-siRNA3). An additional scrambled sequence was also designed as a negative control as follows: 5′-TTCTCCGAACGTGTCACGT-3′ (NC). The lentiviral vectors IL-33 and LV-NC were generated as follows: the oligonucleotides were designed according to the structure of the siRNA sense strand-loop-siRNA antisense strand and were cloned into the lentiviral vector pGCL-GFP (Shanghai Gene Chemical Co., Ltd., Shanghai, China). To generate the lentivirus, the recombinant vector and packaged plasmids were transfected into 293T cells (The Shanghai Research Institute of Chinese Academy of Sciences, Beijing, China). The transfection results were observed under a fluorescence microscope (Leica).

### Transduction of primary cultured astroglial cells and GFP fluorescence observation

Primary astrocytes were prepared from the spinal cords of Sprague-Dawley rats (1–2 days old) (Experimental Animal Center of the Central South University) according to a previous report [26]. Astroglial cells were plated in 6-well dishes coated with poly-d-lysine and laminin (Sigma) and were cultured in Dulbecco’s modified Eagle’s medium (DMEM) (Gibco) supplemented with 10% fetal bovine serum (FBS) (Gibco). Dissociated astroglial cells were infected with lentivirus vectors 1 h after plating at a multiplicity of infection (M.O.I.) = 10 TU/cell, yielding approximately 95% infection of astroglial cells with no apparent toxicity. The expression of green fluorescent protein in the cells was monitored with a fluorescence microscope (Leica) at 72 h post-infection. The positive expression showed the success of the infection process.

### SiRNA transfections

The astroglial cells were divided into five groups, as follows: control, which were incubated in DMEM medium only; LV-NC, which were transfected with NC; and IL-33-siRNA (1–3), which were transfected with the siRNA targeting IL-33. After transfection for 96 h, we collected duplicates of each condition for mRNA and protein analysis. Real-time quantitative polymerase chain reaction (PCR) and Western blot showed that the IL-33 mRNA and protein in the infected rat spinal cord astroglial cells decreased, and the IL-33-siRNA2 group decreased more significantly. Hence, we selected the IL-33-siRNA2 group for the following experiment and referred to it as LV-shIL-33.

### Animals

Male Sprague-Dawley rats were purchased from the Experimental Animal Center of the Central South University. All the animals were specific pathogen-free grade, weighed 260–300 g, and were housed under barrier-sustained conditions at a constant temperature of 24 ± 1 °C (mean ± SEM) with a 12-h light/dark cycle. Food and water were provided ad libitum. All the animal studies were approved by the Animal Care and Use Committee at the Central South University and were in accordance with the university’s guidelines for the care and use of laboratory animals (NO. 201603359). The ethical guidelines from the International Association for the Study of Pain (IASP) were followed [27].

### Intrathecal catheter implantation

Intrathecal catheters were implanted as described by Yaksh and Rudy [28]. Briefly, rats were anesthetized with a mixture of ketamine plus xylazine anesthesia (80:10 mg/kg, intraperitoneally), and the skin was incised over the occipital area. Following displacement of the occipital muscles, the cisterna magna was punctured with a 22-gauge cannula, and then a polyethylene catheter (PE-10) was carefully inserted via an incision in the cisterna magna and was advanced 7.5–8.0 cm caudally to the level of the lumbar enlargement. Correct intrathecal placement was confirmed by the dragging or paralysis of bilateral hind limbs after injection of 10 μl of 2% lidocaine. The incision site was closed in layers, and the catheter was fixed firmly under the skin and sealed effectively. After a 3-day recovery period, all the animals, except those that appeared to have signs of motor weakness or paralysis, were used for the experiments.

### Rat models of noncompressive lumbar disk herniation

Noncompressive lumbar disk herniation rat models were established as previously described [11, 14]. The rats were anesthetized with a mixture of ketamine plus xylazine (80:10 mg/kg, intraperitoneally), and then, a midline dorsal incision was made over the lumbar spine. The multifidus muscles were removed along the L4–L6 spinous processes, and the right L5 spinal nerve root and the DRG were exposed through laminectomy. An autologous NP was harvested from two coccygeal intervertebral disks and was applied on the L5 DRG without mechanical compression. In the sham operated group, the NP was also harvested from the tails, but a similar volume of muscle was harvested from the surgical area on the back and was applied instead to the DRG.

### Protocols

On day 0, a noncompressive lumbar disk herniation rat model was initiated. The control rats underwent the same operation without the application of NP. On day 1, the virus (5 or 10 μl) was administered through the implanted catheter, which was flushed by using 10 or 5 μl of PBS. The rats were spinally delivered with NS, LV-NC, or LV-shIL-33 plus PBS in a total volume of 15 μl. Thus, 90 rats were randomly assigned to 5 groups of 18 rats each, consisting of the sham group, vehicle group, LV-NC group, LV-shIL-33 (5 μl) group, and LV-shIL-33 (10 μl) group.

To confirm the knockdown of IL-33 expression by an intrathecally delivered LV-shIL-33, the ipsilateral spinal dorsal horn tissues (L4–L6) were removed on day 12, and the expression of IL-33 was assayed by real-time quantitative PCR and Western blot analysis (*n* = 6/group). Further experiments on these groups of animals included the assessment of pain-related behavior, cytokine level measurement, and an evaluation of the MAPK and NF-κB pathways (*n* = 4–6/group).

### Real-time quantitative PCR

Total RNA was extracted by the total RNA isolation reagent (Invitrogen), and cDNA was transcribed using a Transcriptor First Strand cDNA Synthesis Kit (Takara), both in accordance with the manufacturer’s instructions. The primers utilized for the PCR were as follows: IL-33, forward primer, 5’-CCCTGAGCACATACAACGACC-3′ and reverse primer, 5’-CACCATCAGCTTCTTCCCATC-3′; and GAPDH (as internal control), forward primer, 5’-TTCAACGGCACAGTCAAGG-3′ and reverse primer, 5’-CTCAGCACCAGCATCACC-3′. The PCR was performed using a 2720 Thermal cycler (Applied Biosystems). The reaction conditions consisted of an initial denaturation step at 95 °C for 10 min, followed by 40 cycles at 95 °C for 15 s, 58 °C for 25 s, and 72 °C for 25 s, and then a final elongation step at 95 °C for 15 s, 60 °C for 1 min, and 95 °C for 15 s. The change in mRNA levels was determined using the 2^−ΔΔCt^ method with normalization software.

### Western blot analysis

Ipsilateral spinal dorsal horn tissues (L4–L6) were homogenized in RIPA buffer (Beyotime) supplemented with 1 m*M* phenylmethyl sulfonylfluoride (Beyotime) on ice and were centrifuged at 16000×*g* for 30 min at 4 °C. The supernatant was collected, and the protein levels in the extracts were determined using the BCA Protein Assay kit (Pierce Biotechnology). Subsequently, the protein samples were electrophoresed on a 10% SDS-PAGE gel and were transferred onto a PVDF membrane (Millipore). After blocking with 5% nonfat milk and a 0.1% Tween-20 in tris buffered saline (TBS) solution for 2 h at room temperature, the membranes were incubated overnight at 4 °C with a rabbit anti-IL-33 antibody (Novus Biologicals), a rabbit anti-ST2 antibody (Proteintech), a rabbit anti-COX-2 antibody (Abcam), a rabbit anti-phospho-ERK antibody (Cell Signaling), a rabbit anti-ERK antibody (Cell Signaling), a rabbit anti-phospho-p38 antibody (Cell Signaling), a rabbit anti-p38 antibody (Cell Signaling), a rabbit anti-phospho-JNK antibody (Cell Signaling), a rabbit anti-JNK antibody (Cell Signaling), a rabbit anti-phospho-NF-κB/p65 antibody (Abcam), or a rabbit anti-NF-κB/p65 antibody (Abcam). The membranes were then washed three times with TBS and were incubated with an anti-rabbit secondary antibody (Santa Cruz Biotechnology) for 1 h at 37 °C. After extensive washing, the protein bands were visualized by an enhanced chemiluminescence assay (Millipore) following the manufacturer’s instructions. The blots were also probed with anti-β-tubulin antibody (Santa Cruz Biotechnology), which served as the loading control.

### Immunostaining of the spinal cords

Immunofluorescence staining or double immunostaining was performed as previously described [29]. Briefly, on day 12, the ipsilateral spinal dorsal horn tissues (L4–L6) were harvested and cryosectioned at 10 μm. The tissue sections were fixed in 4% paraformaldehyde, washed in phosphate buffer saline (PBS), and blocked with blocking solution (10% normal goat serum and 0.3% Triton X-100 in 0.1 M PBS), followed by an incubation with a polyclonal rabbit anti-IL-33 antibody (Novus Biologicals) for 24 h at 4°. Thereafter, the sections were incubated with tetramethylrhodamine isothiocyanate (TRITC)-conjugated goat anti-rabbit secondary antibodies (Cowin Biotech) for 1 h at 37 °C. Double immunofluorescence staining was performed using anti-IL-33 or anti-ST2 antibodies and antibodies specific for neurons (neuronal-specific nuclear protein (NeuN)), astrocytes (glial fibrillary acidic protein (GFAP)), microglia (CD11b [OX-42]), or oligodendrocytes (oligodendrocyte transcription factor 2[Olig-2]). Briefly, the tissue sections were incubated with the appropriate mixtures of the following antibodies for 24 h at 4 °C: rabbit anti-IL-33 and mouse anti-NeuN (Chemicon), mouse anti-GFAP (Santa Cruz Biotechnology), mouse anti-OX-42 (Abcam), or mouse anti-Olig-2 (Millipore). This was followed by an incubation with a mixture of TRITC-conjugated goat anti-rabbit and fluorescein isothiocyanate (FITC)-conjugated goat anti-mouse secondary antibodies (CoWin Biotech) for 1 h at 37°. The mean fluorescence intensity and positively stained elements were determined using ImageJ software [30].

### Assessment of pain-related behavior

The mechanical thresholds (paw withdrawal threshold (PWT)) induced by CFA were evaluated with von Frey filaments (Stoelting) by using the “up-down” method, as described in detail previously [31]. The tests were performed at different times (1, 3, 5, 7, 9, 11, 13, 15, 17, 19, and 21 days) after the operation. All the groups were evaluated 1 day before the operation to determine the baseline mechanical thresholds.

### Measurement of cytokine levels

The levels of the proinflammatory cytokines IL-1β, IL-6, and TNF-α were determined in the ipsilateral spinal dorsal horn tissues (L4–L6) according to previously described methods [32]. Briefly, on day 12, the segment samples were pooled and homogenized in ice-cold PBS containing 0.05% Tween-20, 0.1 mM PMSF, 0.1 mM benzethonium chloride, 10 mM EDTA, and 20 UI aprotinin A. After centrifugation at 7000*g* at 4 °C for 10 min, the supernatants were stored at − 80 °C for future protein quantification. The cytokine levels were evaluated using an enzyme-linked immunosorbent assay (ELISA) kit (R&D Systems) according to the manufacturer’s recommendations.

### Statistical analysis

All the statistical analyses were performed using SPSS statistical software (version 19.0; SPSS Inc., Chicago, IL, USA). Pain-related behavior was analyzed by two-way analysis of variance (ANOVA) with repeated measures, followed by Bonferroni’s post hoc test. The variance factors for two-way ANOVA were time and group comparisons. The real-time PCR, Western blot, immunofluorescence, and ELISA data were analyzed by a one-way ANOVA followed by a Bonferroni’s post hoc test. The variance factor for the one-way ANOVA was group comparisons. *P* values < 0.05 were considered statistically significant. The values are expressed as the mean ± SEM.

## Results

### Silencing of the IL-33 gene by a lentivirus-mediated delivery of shRNA in astroglial cells in vitro

**Fig. 1:**
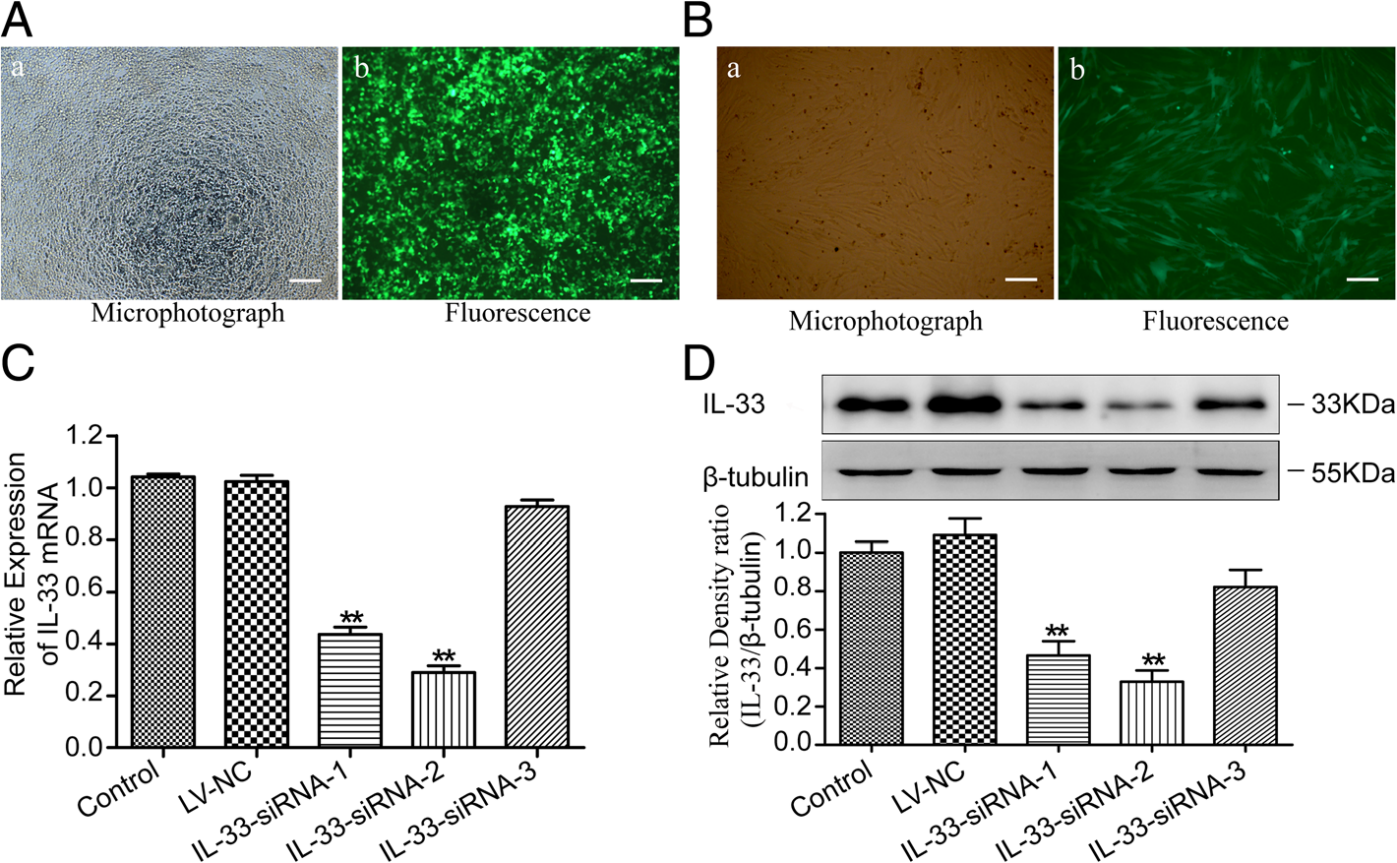
Silencing of the IL-33 gene by lentivirus-mediated delivery of shRNA in astroglial cells in vitro. **a** Fluorescence microscopy showed GFP fluorescence expression in nearly all the cells, which indicated a high and stable transduction of the lentiviral vector system. Bars = 100 μm. **b** Fluorescence microscopy images of astroglial cells 72 h after transduction with LV-shIL-33. Bars = 100 μm. **c** Representative real-time PCR of IL-33 mRNA expression in astrocytes in vitro. The fold change of the IL-33 levels in the control group was set as 1.0 for the quantifications. **d** Representative Western blots of the IL-33 protein expression in astroglial cells in vitro. β-tubulin was used as a loading control. The results are expressed as the ratio of IL-33 to β-tubulin, and the control was set at 1.0. The values are the mean ± SEM (**p < 0.01 versus the LV-NC group or the vehicle group, *n* = 6 in each group)

Fluorescence microscopy showed GFP fluorescence expression in nearly all the cells (Fig. 1a, parts a–b), which indicated a high and stable transduction of the lentiviral vector system. The final titer of the recombinant virus was 5 × 10^8^ transducing units (TU)/ml. In addition, GFP fluorescence was detected in more than 95% of the cultured astroglial cells at 72 h post-infection (Fig. 1b, parts a–b), indicating a successful infection. On the other hand, no cell death and no significant changes in cell morphology were shown, indicating that the lentiviral vector had no toxic effect on the astroglial cells.

Real-time PCR and Western blot analyses were performed 96 h after infection to evaluate the level of IL-33 mRNA and protein expression (Fig. 1c, d). The levels of IL-33 mRNA and protein differed significantly between the groups (for mRNA, F[4,25] = 228.033, *p* < 0.01; for protein, F[4,25] = 15.930, *p* < 0.01). Compared with the LV-NC group or the control group, the IL-33-siRNA-1 and IL-33-siRNA-2 groups showed a trend toward less expression of IL-33 mRNA and protein (overall *p* < 0.01), whereas no significant difference in IL-33 mRNA and protein expression were observed between the control group and the LV-NC group or the IL-33-siRNA-3 group. Moreover, as shown in Fig. 1c, d, the IL-33-siRNA2 group showed a more significant decrease. Thus, we selected the IL-33-siRNA2 group to continue with the following experiment and named the group LV-shIL-33.

### Expressions of interleukin (IL)-33 and its receptor ST2 in the spinal cord in rat models of noncompressive lumbar disk herniation

**Fig. 2:**
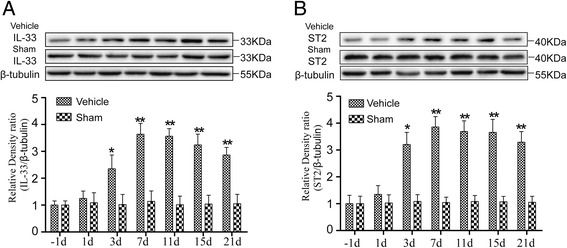
Expressions of interleukin (IL)-33 and its receptor ST2 in the spinal cord in rat models of noncompressive lumbar disk herniation. **a** Western blot showing the time course of the changes in the expression of IL-33 after the operation. β-tubulin was used as a loading control. The results are expressed as the ratio of IL-33 to β-tubulin, and the control was set at 1.0. **b** Western blot showing the time course of the changes in the expression of ST2 after the operation. β-tubulin was used as a loading control. The results are expressed as the ratio of ST2 to β-tubulin, and the control was set at 1.0. The values are the mean ± SEM (##*p* < 0.01 versus sham; **p < 0.01 versus the LV-NC group or the vehicle group, *n* = 4–5 in each group)

**Fig. 3:**
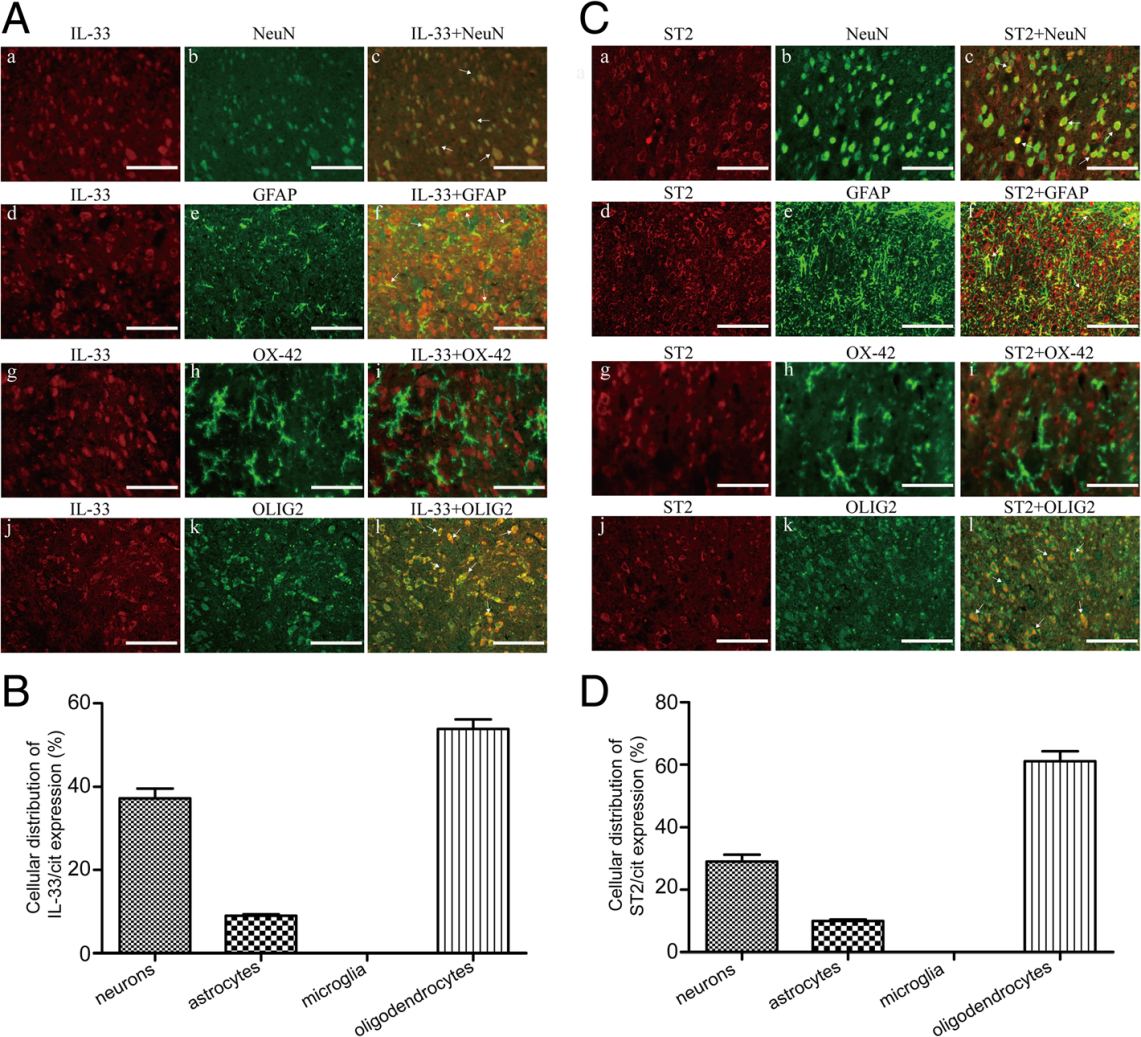
Cellular localization of IL-33 and ST2 in the rat ipsilateral spinal dorsal horn. **a** Cell type-specific immunolabeling of IL-33 in the ipsilateral spinal dorsal horn tissue shows colocalization of IL-33 immunoreactivity (in red) with NeuN-immunoreactive neurons (in green; panels **a**–**c**), GFAP-immunoreactive astrocytes (in green; panels **d**–**f**), and olig-2-immunoreactive oligodendrocytes (in green; panels **j**–**l**), but not with OX-42-immunoreactive microglia (in green; panels **g**–**i**). The arrows indicate the colocalization of IL-33 with the respective cell markers (in yellow). Bars = 50 μm. **b** The percentage of the cell types expressing IL-33/cit is presented. **c** Cell type-specific immunolabeling of ST2 in the ipsilateral spinal dorsal horn tissue shows colocalization of ST2 immunoreactivity (in red) with NeuN-immunoreactive neurons (in green; panels **a**–**c**), GFAP-immunoreactive astrocytes (in green; panels **d**–**f**), and olig-2-immunoreactive oligodendrocytes (in green; panels **j**–**l**), but not with OX-42-immunoreactive microglia (in green; panels **g**–**i**). The arrows indicate the colocalization of ST2 with the respective cell markers (in yellow). Bars = 50 μm. **d** The percentage of cellular types expressing ST2/cit is presented

To explore the expression of IL-33 and ST2 in the ipsilateral spinal dorsal horns (L4–L6) in rat models of noncompressive lumber disk herniation, we conducted Western blot analysis. The expression of spinal IL-33 and ST2 was significantly increased by the third day, peaked at the 7th day and was maintained at a high level until the final examination on the 21st day after the operation (Fig. 2a, b). Moreover, to determine which cell types express IL-33 and ST2 in a rat model of noncompressive lumbar disk herniation, double staining was performed using anti-IL-33 or anti-ST2 antibodies and antibodies specific for neurons (NeuN), astrocytes (GFAP), microglia (OX-42), or oligodendrocytes (Olig-2). IL-33 and ST2 localized in the neurons, astrocytes, and oligodendrocytes, as it colocalized with the neuronal marker NeuN, the astrocytic marker GFAP, and the oligodendrocytic marker Olig-2, respectively (for IL-33, Fig. 3a, parts a–f and parts j–l; for ST2, Fig. 3c, parts a–f and parts j–l). In contrast, IL-33 and ST2 did not colocalize with the microglial marker OX-42 (for IL-33, Fig. 3a, parts g–i; for ST2, Fig. 3c, parts g–i), indicating that IL-33 and ST2 were not expressed in microglia. Moreover, the main IL-33/cit-expressing cells in the spinal dorsal horn were oligodendrocytes and neurons. Low expression of IL-33/cit was found in cells expressing GFAP (for IL-33, Fig. 3b; for ST2, Fig. 3d).

### Spinally delivered LV-shIL-33 inhibits IL-33 expression in the dorsal spinal cord in vivo

**Fig. 4:**
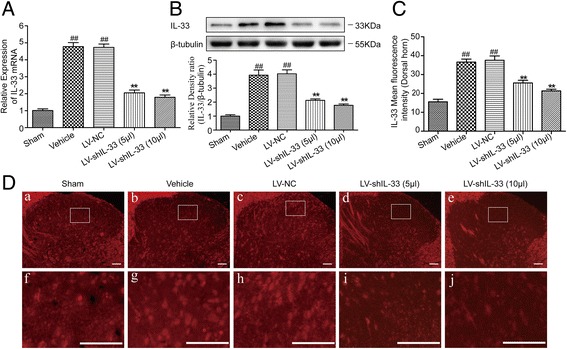
Spinally delivered lentiviral vector LV-shIL-33 inhibits the expression of IL-33 in the dorsal spinal cord. **a** Representative real-time PCR of IL-33 mRNA expression in the rat ipsilateral spinal dorsal horns tissue. The fold change of IL-33 levels in the sham group was set as 1.0 for the quantifications (*n* = 6 in each group). **b** Representative Western blots of the IL-33 protein expression in the rat ipsilateral spinal dorsal horn tissue. β-tubulin was used as a loading control. The results are expressed as the ratio of IL-33 to β-tubulin, and the control was set at 1.0 (*n* = 6 in each group). **c** Quantification of immunofluorescence signals for IL-33 in the rat ipsilateral spinal dorsal horns (*n* = 4–5 in each group). **d** Representative immunofluorescence detection of IL-33 in the rat ipsilateral spinal dorsal horns. Panels **f**–**j** are higher-power magnifications of the boxed areas in the panels **a**–**e**. Bars = 50 μm (*n* = 6 in each group). The values are the mean ± SEM (^##^*p* < 0.01 versus sham; **p < 0.01 versus the LV-NC group or the vehicle group)

On the 12th day after the operation, the real-time PCR and Western blot were utilized to determine the IL-33 expression changes in the ipsilateral spinal dorsal horns (L4–L6). As shown in Fig. 4a, b, the levels of IL-33 mRNA and protein differed significantly between the groups (for mRNA, F[4,25] = 101.186, *p* < 0.01; for protein, F[4,25] = 56.382, *p* < 0.01). Bonferroni’s post hoc test revealed that the expression of IL-33 at the mRNA and protein levels in the vehicle group was markedly increased compared with those in the sham group (for mRNA, *p* < 0.01; for protein, *p* < 0.01), whereas no significant difference in IL-33 mRNA and protein expression was observed between the vehicle group and the LV-NC group. Moreover, compared with the LV-NC group or the vehicle group, the LV-shIL-33 (5 μl) and LV-shIL-33 (10 μl) groups showed a trend of less expression of IL-33 mRNA and protein (overall *p* < 0.01). In addition, immunostaining was performed to detect the expression of IL-33 in the spinal dorsal horn (Fig. 4c, d). Compared with the sham group, the rats in the vehicle group exhibited a significantly higher IL-33 expression, as demonstrated by the intense red fluorescence localized in the nerve cells (*p* < 0.01). Moreover, spinally delivered LV-shIL-33 (5 or 10 μl) markedly decreased the expression of IL-33 in the spinal dorsal horn (overall *p* < 0.01).

### Effects of spinally delivered LV-shIL-33 on mechanical allodynia

**Fig. 5:**
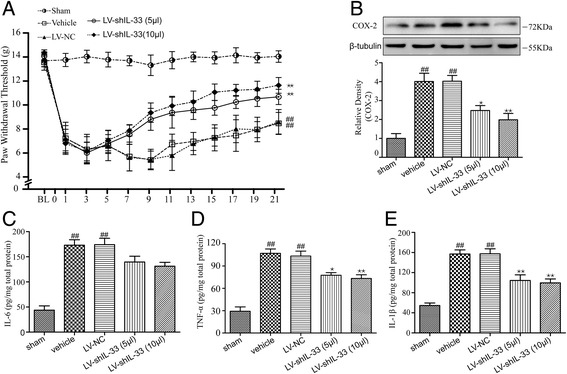
Spinally delivered lentiviral vector LV-shIL-33 alleviates mechanical allodynia and decreases the production of spinal IL-1β, TNF-α, and COX-2, but not IL-6 in rat models of noncompressive lumbar disk herniation. **a** Paw withdrawal threshold was performed 1 day before the operation (baseline (BL)) and at different times (1, 3, 5, 7, 9, 11, 13, 15, 17, 19, and 21 days) after the operation. The values are the mean ± SEM (^##^*p* < 0.01 versus sham; **p < 0.01 versus the LV-NC group or the vehicle group, *n* = 10 in each group). **b** The expression levels of spinal COX-2 were assessed at the protein level by Western blotting 12 days after the operation. β-tubulin was used as the loading control. The results are expressed as the ratio of COX-2 to β-tubulin, and the control was set at 1.0. (**c**, **d**, and **e**). The expression levels of spinal IL-1β (**c**), IL-6 (**d**), and TNF-α (**e**) were assessed at the protein level by ELISA in each treatment group 12 days after the operation. The values are the mean ± SEM (^##^*p* < 0.01 versus sham; *p < 0.05 and **p < 0.01 versus the LV-NC group, *n* = 5–6 in each group)

The paw withdrawal thresholds in response to mechanical stimuli decreased significantly (*p* < 0.01 versus sham) after the application of NP to the L5 DRG from day 1 to the last time point assessed (day 21), whereas no trend toward a differential response was found between the vehicle group and the LV-NC group. Moreover, the mechanical allodynia of the animals in the LV-shIL-33 (5 μl) and LV-shIL-33 (10 μl) groups was markedly higher than that of the animals in the LV-NC group or vehicle group (each *p* < 0.01) (Fig. 5a).

### Effects of spinally delivered LV-shIL-33 on the expression of IL-1β, IL-6, TNF-α, and COX-2 in the spinal cord

We observed that the levels of IL-1β, IL-6, and TNF-α differed markedly between the groups (for IL-β: F[4,25] = 25.430, *p* < 0.01; for IL-6: F[4,25] = 26.176, *p* < 0.01; for TNF-α: F[4,25] = 32.807, *p* < 0.01) (Fig. 5c–e). The results of the Bonferroni post hoc test indicated that the expression levels of IL-1β, IL-6, and TNF-α were all significantly increased in the vehicle group compared with the sham group (for IL-1β: *p* < 0.01; for IL-6: *p* < 0.01; for TNF-α: *p* < 0.01), whereas no significant difference in the expression levels of IL-1β, IL-6, and TNF-α was observed between the vehicle group and the LV-NC group. In addition, the IL-1β and TNF-α protein levels in the LV-shIL-33 (5 μl) and LV-shIL-33 (10 μl) groups were significantly lower than those in the LV-NC group (for IL-1β: each *p* < 0.01; for TNF-α: LV-shIL-33 (5 μl), *p* < 0.05; LV-shIL-33 (10 μl), *p* < 0.01), whereas the expression of IL-6 was modestly but not significantly lower in the LV-shIL-33 (5 μl) and LV-shIL-33 (10 μl) groups (LV-shIL-33 (5 μl), *p* = 0.273; LV-shIL-33 (10 μl), *p* = 0.074).

We also evaluated the effect of spinally delivered LV-shIL-33 on spinal COX-2 expression in the rat models of noncompressive lumbar disk herniation. As shown in Fig. 5b, the levels of spinal COX-2 protein differed significantly between the groups (F[4,20] = 16.323, *p* < 0.01), and the levels were markedly increased in the vehicle group (*p* < 0.01). Moreover, compared with the LV-NC group, the level of COX-2 was significantly lower in the LV-shIL-33 (5 μl) and LV-shIL-33 (10 μl) groups (LV-shIL-33 (5 μl), *p* < 0.05; LV-shIL-33 (10 μl), *p* < 0.01).

### Effects of spinally delivered LV-shIL-33 on activation of the MAPK and NF-κB pathways

**Fig. 6:**
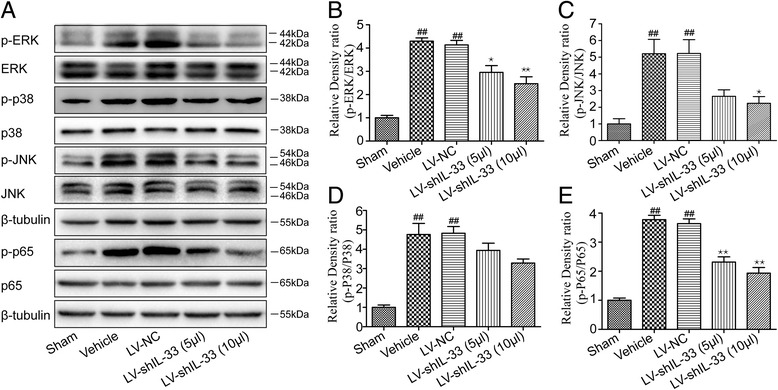
Spinally delivered lentiviral vector LV-shIL-33 reduces the activation of the MAPK and NF-κB pathways in the dorsal spinal cord. **a** Representative Western blots of the phosphorylation of ERK, p38, JNK, and p65 protein expression in the rat ipsilateral spinal dorsal horn tissue. β-tubulin was used as the loading control. **b** The results are expressed as a ratio of phospho-protein to the total protein (p-ERK/ERK), and the control was set at 1.0. **c** The results are expressed as a ratio of phospho-protein to total protein (p-JNK/JNK), and the control was set at 1.0. **d** The results are expressed as a ratio of phospho-protein to total protein (p-p38/p38), and the control was set at 1.0. **e** The results are expressed as a ratio of phospho-protein to total protein (p-p65/p65), and the control is set at 1.0. The values are the mean ± SEM (^##^*p* < 0.01 versus sham; *p < 0.05 and **p < 0.01 versus the LV-NC group, *n* = 5 in each group)

To determine whether IL-33 influences the activation of the MAPK pathways, the expression of p-ERK, p-p38, p-JNK, ERK, p38, and JNK was measured. As shown in Fig. 6a–d, the level of p-ERK, p-p38, and p-JNK differed markedly between the groups (for p-ERK: F[4,20] = 37.305, *p* < 0.01; for p-JNK: F[4,20] = 9.657, *p* < 0.01; for p-p38: F[4,20] = 19.183, *p* < 0.01), but the levels of ERK, p38, and JNK protein did not change. The results of Bonferroni’s post hoc test indicated that the levels of p-ERK, p-p38, and p-JNK were all significantly elevated in the vehicle group compared with the sham group (each *p* < 0.01), whereas no significant difference in the levels of p-ERK, p-p38, and p-JNK were observed between the vehicle group and the LV-NC group. In addition, when compared with the LV-NC group, the level of p-ERK was significantly lower in the LV-shIL-33 (5 μl) and LV-shIL-33 (10 μl) groups (LV-shIL-33 (5 μl), *p* < 0.05; LV-shIL-33 (10 μl), *p* < 0.01). Moreover, compared with the LV-NC group, the LV-shIL-33 group (10 μl) showed decreased expression of p-JNK protein (*p* < 0.05), while the LV-shIL-33 (5 μl) group showed modestly reduced expression of p-JNK protein; the difference did not reach statistical significance (*p* = 0.071). Furthermore, the level of p-p38 was not statistically significantly different between the LV-NC group and the LV-shIL-33 (5 μl) or LV-shIL-33 (10 μl) groups.

To determine whether IL-33 influences expression of the NF-κB pathways, the phosphorylation of NF-κB/p65 was measured (Fig. 6a, e). We observed that the level of p-p65 markedly differed between the groups (F[4,20] = 54.774, *p* < 0.01). In addition, markedly higher levels of p-p65 in the spinal dorsal horns were exhibited in the vehicle group when compared with the sham group. Moreover, p-p65 expression levels in the LV-shIL-33 (5 μl) and LV-shIL-33 (10 μl) groups were obviously lower than those in the LV-NC group (overall *p* < 0.01).

## Discussion

Radicular pain caused by intervertebral disk herniation was initially considered to result from mechanical compression of the nerve roots by a herniated NP [3–5]. However, accumulating evidence indicates that radicular pain results from inflammation of the nerve roots, alone or combined with mechanical compression [6–8]. Radiculopathic symptoms are not always present with nerve root compression and can occur in its absence [7, 8]. In animal models, the direct application of NP to the nerve roots without mechanical compression causes an abnormal production of inflammatory mediators and cytokines [10, 13, 33–38], which rapidly sensitize primary afferents and induce neural-immune interactions [39, 40]. This leads to the production and secretion of cytokines, excitatory amino acids, COX-2, and prostaglandins, which induces the hyperexcitability of spinal dorsal horn neurons as well as hyperalgesia [41, 42].

In the current study, our results indicated the application of NP to the L5 DRG induced an increase in IL-33 and ST2 expression in the spinal dorsal horn. This finding supports the idea that spinal IL-33/ST2 signaling may be among the important proteins that are activated by protuberant NP and are, thus, involved in the development of hyperalgesia. Previous studies demonstrate that a peripheral injection of IL-33 induces cutaneous hypernociception and involves in the modulation of arthritic pain [21]. It is also reported that the IL-33/ST2 signaling plays a pivotal role in mediating acute inflammatory pain by using a mouse model of formalin-induced inflammatory pain [20]. In addition, recent studies have proven that spinal IL-33/ST2 signaling plays a key role in the modulation of neuropathic pain and chronic bone cancer pain [22, 23, 43]. Here, we showed that a spinally delivered inhibitor of IL-33 expression, the lentiviral vector LV-shIL-33, significantly attenuated the mechanical allodynia in rat models of noncompressive lumbar disc herniation. Taken together, these results suggested that IL-33/ST2 signaling participates in both acute and chronic pain, including radicular, arthritic, neuropathic, and bone cancer pain.

IL-33 has been previously reported to be co-expressed in both astrocytes and neurons but not in microglia in the murine spinal dorsal horn [44]. Moreover, a recent study showed that the main cells expressing IL-33 are oligodendrocytes rather than neurons and astrocytes [23]. Similarly, our findings showed that IL-33-like immunoreactivity was mainly localized in the rat spinal neurons, astrocytes, and oligodendrocytes. In addition, previous studies report that IL-33 is predominantly localized in astrocytes, but not in microglia or neurons in the mouse brain and spinal cord [22, 43, 45], providing conflicting findings regarding the true distribution of IL-33. Similar to IL-33, the expression of ST2 in the CNS is controversial. Some reports show that ST2 is expressed in astrocytes [45], whereas others demonstrate its expression in both astrocytes and neurons in the murine spinal cord [22, 43]. Our results showed that ST2 was distributed in the astrocytes, neurons, and oligodendrocytes in the spinal dorsal horn in rat. These results are inconsistent and may be due to the different central nervous system regions studied, the different antibodies used, or the differences between mouse and rat.

It has been reported that the upregulation of the spinal *N*-methyl-*D*-aspartate (NMDA) receptor subunit 1 after spared nerve injury is reduced by ST2 antibody administration or ST2−/−. The induction of nociceptive behaviors in naive mice due to recombinant IL-33 is reversed by the noncompetitive NMDA antagonist MK-801 [22]. In addition, ST2 antibody administration or ST2−/− markedly inhibits the increased activation of the astroglial janus kinase 2 (JAK2)-signal transducer and activator of transcription 3 (STAT3) cascade and the neuronal calcium–calmodulin-dependent kinase II (CaMKII)-cyclic adenosine monophosphate response element-binding protein (CREB) cascade after spared nerve injury. Moreover, a recent study demonstrated that oligodendrocyte-derived IL-33 plays an important role in neuropathic pain [23]. Therefore, IL-33 upregulation in the spinal cord after peripheral nerve injury may reflect changes in neuron, astrocyte, and oligodendrocytes function.

Moreover, we found that the expression of spinal IL-1β, IL-6, TNF-α, and COX-2 protein was highly upregulated in rat models of noncompressive lumber disk herniation. In the family of proinflammatory cytokines, IL-1β, IL-6, and TNF-α have long been strongly implicated in the modulation of peripheral pain and the inflammatory process induced by the contact of the NP with the nerve fibers [10, 13, 33, 37]. In addition to their peripheral action, IL-1β, IL-6, and TNF-α are upregulated in the spinal cord in rat models of lumber disk herniation, and all are known to be involved in the development and maintenance of radicular pain caused by intervertebral disc herniation [6, 8–10]. COX-2, which is constitutively expressed in the spinal cord, is a major contributor to the elevation of spinal prostaglandin E2 [46]. It has been shown that lumbar disk herniation to the spinal nerve root upregulates the expression of COX-2 in the spinal dorsal and ventral horns, and an intrathecal administration of an antibody to COX-2 attenuates hyperalgesia [14]. In addition, a previous study suggests that IL-33 plays an essential role in bone cancer pain, and its actions might be associated with inducing spinal upregulation of IL-1β and TNF-α. Moreover, recent evidence indicated that spinal IL-33-mediated hyperalgesia during chronic constriction injury (CCI) is dependent on a reciprocal relationship with TNF-α and IL-1β [23]. Similarly, our results indicated that spinally delivered LV-shIL-33 markedly reduced the overexpression of spinal IL-1β, TNF-α, and COX-2 in rat models of noncompressive lumber disk herniation. These findings indicated that spinal IL-33 plays an important role in the regulation of IL-1β, TNF-α, and COX-2 expression in the spinal cord and is involve in the modulation of radicular pain.

The underlying mechanism of IL-33 involved in neuroinflammatory and hyperalgesia is still under investigation. The activation of the MAPK and NF-κB pathways might be mediated. The MAPK pathway participates in central sensitization and nociceptive specific signaling in dorsal horn neurons [47]. A remarkable increase in the level of p-ERK and p-JNK was detected in the neuropathic pain model of rats, and an intrathecal administration of p-ERK or p-JNK inhibitors significantly attenuated the pain behavior [48, 49]. Reports also show that the expression of p-ERK and p-JNK increase in the spinal cord in rat models of lumbar disc herniation [7, 15, 50]. In addition, a previous report indicated that lumbar disk herniation to the spinal nerve root increases the expression of p-p38 in the spinal cord microglia, and an intrathecal injection of p38 inhibitors significantly attenuates hyperalgesia [51]. Moreover, a recent study revealed that after formalin injection, the expression of p-ERK, p-p38, and p-JNK are remarkably increased in the spinal cord, and the inhibition of spinal interlukin-33/ST2 signaling attenuates the expression of p-ERK and p-JNK in the spinal cord [52]. Similar to the findings in these studies, the present study showed that the application of NP to the L5 DRG induced an upregulation in the expression of phosphorylated components of the MAPK pathway, including p-ERK, p-JNK, and p-p38 in the spinal cord, and a spinally delivered inhibitor of IL-33 expression, the lentiviral vector LV-shIL-33, significantly alleviated the activation of p-JNK and p-ERK, but not p-p38 in the spinal dorsal horn in rat models of noncompressive lumber disk herniation. It is reported that ERK and JNK are mainly distributed and activated in the astrocytes and neurons, whereas p38 MAPK is mainly distributed and activated in microglia [47, 52]. Moreover, we found that ST2 is mainly distributed in the neurons, astrocytes, and oligodendrocytes and is not in the microglia in the spinal cord. Thus, we speculate that IL-33 is released from the astrocytes and oligodendrocytes and then acts on the astroglial or neuronal ST2 receptor in an autocrine or paracrine manner during radicular pain. We, therefore, propose that the analgesia effect and regulation of the inflammatory mediator of LV-shIL-33 might occur partially by inhibiting the ERK and JNK pathways in spinal astrocytes or neurons but not by activating microglial p-38 MAPK.

The NF-κB family of transcription factors controls the expression of genes that are critical for inflammation and the immune process. Our previous study indicated an extensive colocalization of NF-κB/p65 with TNF-α in the ipsilateral spinal dorsal horn of the CCI rat model, and a downregulation of spinal NF-κB/p65 expression markedly decreased sciatic nerve ligation-induced mechanical and thermal hyperalgesia. Moreover, previous studies indicate that the activation of NF-κB is significantly increased in the DRG and spinal cord in rat models of lumber disk herniation [8, 53], and an intrathecal injection of an NF-κB decoy markedly decreases mechanical allodynia and thermal hyperalgesia induced by protuberant NP [53]. Furthermore, in adjuvant-induced arthritis (AIA) rats, we previously observed that spinal NF-κB/p65 is mainly distributed in the dorsal horn neurons and astrocytes, and it plays a critical role in the initiation and development of both peripheral inflammation and inflammation-related hyperalgesia by regulating the expression of inflammatory mediators, such as TNF-α, IL-1β, and COX-2 [54]. In the current study, we detected a significant attenuation of mechanical allodynia and a decrease of inflammatory mediators after the spinal injection of LV-shIL-33, which might be associated with the inhibition of NF-κB/p65 in the spinal dorsal horn.

## Conclusions

Our findings indicate that the analgesic effect of LV-shIL-33 might be mediated at least in part by the prevention of neuronal, astrocytic, and oligodendrocytic IL-33 expression and the subsequent downregulation of inflammatory mediators and inhibition of the ERK, JNK, and NF-κB/p65 pathways. Thus, these findings may bring new evidence for a novel treatment option of radicular pain caused by intervertebral disk herniation.

## Abbreviations

COX-2: Cyclooxygenase 2
ERK: Extracellular signal-regulated kinase
GFAP: Glial fibrillary acidic protein
IL-1β: Interleukin-1β
IL-33: Interleukin-33
IL-6: Interleukin-6
JNK: C-Jun N-terminal kinase
NeuN: Neuronal-specific nuclear protein
NF-κB: Nuclear factor-κB
NO: Nitric oxide
NOS: Nitric oxide synthase
NP: Nucleus pulposus
PCR: Polymerase chain reaction
siRNAs: Small interfering RNAs DMEM: dulbecco’s modified eagle medium
TNF: Tumor necrosis factor

## References

[1] Hoy D, March L, Brooks P, Blyth F, Woolf A, Bain C, Williams G, Smith E, Vos T, Barendregt J (2014). The global burden of low back pain: estimates from the global burden of disease 2010 study. Ann Rheum Dis.

[2] Younes M, Bejia I, Aguir Z, Letaief M, Hassen-Zrour S, Touzi M, Bergaoui N (2006). Prevalence and risk factors of disk-related sciatica in an urban population in Tunisia. Joint Bone Spine.

[3] Winkelstein BA, Weinstein JN, DeLeo JA (2002). The role of mechanical deformation in lumbar radiculopathy: an in vivo model. Spine (Phila Pa 1976).

[4] Tabo E, Jinks SL, Eisele JH, Carstens E (1999). Behavioral manifestations of neuropathic pain and mechanical allodynia, and changes in spinal dorsal horn neurons, following L4-L6 dorsal root constriction in rats. Pain.

[5] Konno S, Yabuki S, Sato K, Olmarker K, Kikuchi S (1995). A model for acute, chronic, and delayed graded compression of the dog cauda equina. Presentation of the gross, microscopic, and vascular anatomy of the dog cauda equina and accuracy in pressure transmission of the compression model. Spine (Phila Pa 1976).

[6] Molinos M, Almeida CR, Caldeira J, Cunha C, Goncalves RM, Barbosa MA (2015). Inflammation in intervertebral disc degeneration and regeneration. J R Soc Interface.

[7] Miao GS, Liu ZH, Wei SX, Luo JG, Fu ZJ, Sun T (2015). Lipoxin A4 attenuates radicular pain possibly by inhibiting spinal ERK, JNK and NF-kappaB/p65 and cytokine signals, but not p38, in a rat model of non-compressive lumbar disc herniation. Neuroscience.

[8] Liu ZH, Miao GS, Wang JN, Yang CX, Fu ZJ, Sun T (2016). Resolvin D1 inhibits mechanical hypersensitivity in sciatica by modulating the expression of nuclear factor-kappaB, phospho-extracellular signal-regulated kinase, and pro- and antiinflammatory cytokines in the spinal cord and dorsal root ganglion. Anesthesiology.

[9] Kawakami M, Matsumoto T, Kuribayashi K, Tamaki T (1999). mRNA expression of interleukins, phospholipase A2, and nitric oxide synthase in the nerve root and dorsal root ganglion induced by autologous nucleus pulposus in the rat. J Orthop Res.

[10] Burke JG, Watson RW, McCormack D, Dowling FE, Walsh MG, Fitzpatrick JM (2002). Intervertebral discs which cause low back pain secrete high levels of proinflammatory mediators. J Bone Joint Surg (Br).

[11] Kim SJ, Park SM, Cho YW, Jung YJ, Lee DG, Jang SH, Park HW, Hwang SJ, Ahn SH (2011). Changes in expression of mRNA for interleukin-8 and effects of interleukin-8 receptor inhibitor in the spinal dorsal horn in a rat model of lumbar disc herniation. Spine (Phila Pa 1976).

[12] Cuellar JM, Montesano PX, Carstens E (2004). Role of TNF-alpha in sensitization of nociceptive dorsal horn neurons induced by application of nucleus pulposus to L5 dorsal root ganglion in rats. Pain.

[13] Le Maitre CL, Hoyland JA, Freemont AJ (2007). Catabolic cytokine expression in degenerate and herniated human intervertebral discs: IL-1beta and TNFalpha expression profile. Arthritis Res Ther.

[14] Ohtori S, Takahashi K, Aoki Y, Doya H, Ozawa T, Saito T, Moriya H (2004). Spinal neural cyclooxygenase-2 mediates pain caused in a rat model of lumbar disk herniation. J Pain.

[15] Schmitz J, Owyang A, Oldham E, Song Y, Murphy E, McClanahan TK, Zurawski G, Moshrefi M, Qin J, Li X (2005). IL-33, an interleukin-1-like cytokine that signals via the IL-1 receptor-related protein ST2 and induces T helper type 2-associated cytokines. Immunity.

[16] Verri WA, Souto FO, Vieira SM, Almeida SC, Fukada SY, Xu D, Alves-Filho JC, Cunha TM, Guerrero AT, Mattos-Guimaraes RB (2010). IL-33 induces neutrophil migration in rheumatoid arthritis and is a target of anti-TNF therapy. Ann Rheum Dis.

[17] Kobori A, Yagi Y, Imaeda H, Ban H, Bamba S, Tsujikawa T, Saito Y, Fujiyama Y, Andoh A (2010). Interleukin-33 expression is specifically enhanced in inflamed mucosa of ulcerative colitis. J Gastroenterol.

[18] Kempuraj D, Twait EC, Williard DE, Yuan Z, Meyerholz DK, Samuel I (2013). The novel cytokine interleukin-33 activates acinar cell proinflammatory pathways and induces acute pancreatic inflammation in mice. PLoS One.

[19] Han P, Mi WL, Wang YQ (2011). Research progress on interleukin-33 and its roles in the central nervous system. Neurosci Bull.

[20] Zarpelon AC, Cunha TM, Alves-Filho JC, Pinto LG, Ferreira SH, McInnes IB, Xu D, Liew FY, Cunha FQ, Verri WA (2013). IL-33/ST2 signalling contributes to carrageenin-induced innate inflammation and inflammatory pain: role of cytokines, endothelin-1 and prostaglandin E2. Br J Pharmacol.

[21] Verri WA, Guerrero AT, Fukada SY, Valerio DA, Cunha TM, Xu D, Ferreira SH, Liew FY, Cunha FQ (2008). IL-33 mediates antigen-induced cutaneous and articular hypernociception in mice. Proc Natl Acad Sci U S A.

[22] Liu S, Mi WL, Li Q, Zhang MT, Han P, Hu S, Mao-Ying QL, Wang YQ (2015). Spinal IL-33/ST2 signaling contributes to neuropathic pain via neuronal CaMKII-CREB and astroglial JAK2-STAT3 cascades in mice. Anesthesiology.

[23] Zarpelon AC, Rodrigues FC, Lopes AH, Souza GR, Carvalho TT, Pinto LG, Xu D, Ferreira SH, Alves-Filho JC, McInnes IB (2016). Spinal cord oligodendrocyte-derived alarmin IL-33 mediates neuropathic pain. FASEB J.

[24] Sun T, Luo J, Jia M, Li H, Li K, Fu Z (2012). Small interfering RNA-mediated knockdown of NF-kappaBp65 attenuates neuropathic pain following peripheral nerve injury in rats. Eur J Pharmacol.

[25] Ui-Tei K, Naito Y, Takahashi F, Haraguchi T, Ohki-Hamazaki H, Juni A, Ueda R, Saigo K (2004). Guidelines for the selection of highly effective siRNA sequences for mammalian and chick RNA interference. Nucleic Acids Res.

[26] Kerstetter AE, Miller RH (2012). Isolation and culture of spinal cord astrocytes. Methods Mol Biol.

[27] Zimmermann M (1983). Ethical guidelines for investigations of experimental pain in conscious animals. Pain.

[28] Yaksh TL, Rudy TA (1976). Chronic catheterization of the spinal subarachnoid space. Physiol Behav.

[29] Boyle DL, Jones TL, Hammaker D, Svensson CI, Rosengren S, Albani S, Sorkin L, Firestein GS (2006). Regulation of peripheral inflammation by spinal p38 MAP kinase in rats. PLoS Med.

[30] O'Brien DE, Alter BJ, Satomoto M, Morgan CD, Davidson S, Vogt SK, Norman ME, Gereau GB, Demaro JA, Landreth GE (2015). ERK2 alone drives inflammatory pain but cooperates with ERK1 in sensory neuron survival. J Neurosci.

[31] Chaplan SR, Bach FW, Pogrel JW, Chung JM, Yaksh TL (1994). Quantitative assessment of tactile allodynia in the rat paw. J Neurosci Methods.

[32] Manjavachi MN, Quintao NL, Campos MM, Deschamps IK, Yunes RA, Nunes RJ, Leal PC, Calixto JB (2010). The effects of the selective and non-peptide CXCR2 receptor antagonist SB225002 on acute and long-lasting models of nociception in mice. Eur J Pain.

[33] Cuellar JM, Borges PM, Cuellar VG, Yoo A, Scuderi GJ, Yeomans DC (2013). Cytokine expression in the epidural space: a model of noncompressive disc herniation-induced inflammation. Spine (Phila Pa 1976).

[34] Park JB, Chang H, Kim YS (2002). The pattern of interleukin-12 and T-helper types 1 and 2 cytokine expression in herniated lumbar disc tissue. Spine (Phila Pa 1976).

[35] Shamji MF, Setton LA, Jarvis W, So S, Chen J, Jing L, Bullock R, Isaacs RE, Brown C, Richardson WJ (2010). Proinflammatory cytokine expression profile in degenerated and herniated human intervertebral disc tissues. Arthritis Rheum.

[36] Jiang H, Deng Y, Wang T, Ma J, Li P, Tian P, Han C, Ma X (2016). Interleukin-23 may contribute to the pathogenesis of lumbar disc herniation through the IL-23/IL-17 pathway. J Orthop Surg Res.

[37] Weiler C, Nerlich AG, Bachmeier BE, Boos N (2005). Expression and distribution of tumor necrosis factor alpha in human lumbar intervertebral discs: a study in surgical specimen and autopsy controls. Spine (Phila Pa 1976).

[38] Miyamoto H, Saura R, Doita M, Kurosaka M, Mizuno K (2002). The role of cyclooxygenase-2 in lumbar disc herniation. Spine (Phila Pa 1976).

[39] Liu B, Li H, Brull SJ, Zhang JM (2002). Increased sensitivity of sensory neurons to tumor necrosis factor alpha in rats with chronic compression of the lumbar ganglia. J Neurophysiol.

[40] Zhang JM, Li H, Liu B, Brull SJ (2002). Acute topical application of tumor necrosis factor alpha evokes protein kinase A-dependent responses in rat sensory neurons. J Neurophysiol.

[41] Anzai H, Hamba M, Onda A, Konno S, Kikuchi S (2002). Epidural application of nucleus pulposus enhances nociresponses of rat dorsal horn neurons. Spine (Phila Pa 1976).

[42] Moalem G, Tracey DJ (2006). Immune and inflammatory mechanisms in neuropathic pain. Brain Res Rev.

[43] Zhao J, Zhang H, Liu SB, Han P, Hu S, Li Q, Wang ZF, Mao-Ying QL, Chen HM, Jiang JW (2013). Spinal interleukin-33 and its receptor ST2 contribute to bone cancer-induced pain in mice. Neuroscience.

[44] Jiang HR, Milovanovic M, Allan D, Niedbala W, Besnard AG, Fukada SY, Alves-Filho JC, Togbe D, Goodyear CS, Linington C (2012). IL-33 attenuates EAE by suppressing IL-17 and IFN-gamma production and inducing alternatively activated macrophages. Eur J Immunol.

[45] Yasuoka S, Kawanokuchi J, Parajuli B, Jin S, Doi Y, Noda M, Sonobe Y, Takeuchi H, Mizuno T, Suzumura A (2011). Production and functions of IL-33 in the central nervous system. Brain Res.

[46] Daher JB, Tonussi CR (2003). A spinal mechanism for the peripheral anti-inflammatory action of indomethacin. Brain Res.

[47] Ji RR, Gereau RW, Malcangio M, Strichartz GR (2009). MAP kinase and pain. Brain Res Rev.

[48] Zhuang ZY, Gerner P, Woolf CJ, Ji RR (2005). ERK is sequentially activated in neurons, microglia, and astrocytes by spinal nerve ligation and contributes to mechanical allodynia in this neuropathic pain model. Pain.

[49] Zhuang ZY, Wen YR, Zhang DR, Borsello T, Bonny C, Strichartz GR, Decosterd I, Ji RR (2006). A peptide c-Jun N-terminal kinase (JNK) inhibitor blocks mechanical allodynia after spinal nerve ligation: respective roles of JNK activation in primary sensory neurons and spinal astrocytes for neuropathic pain development and maintenance. J Neurosci.

[50] Doya H, Ohtori S, Takahashi K, Aoki Y, Ino H, Takahashi Y, Moriya H, Yamashita T (2005). Extracellular signal-regulated kinase mitogen-activated protein kinase activation in the dorsal root ganglion (DRG) and spinal cord after DRG injury in rats. Spine (Phila Pa 1976).

[51] Ito T, Ohtori S, Inoue G, Koshi T, Doya H, Ozawa T, Saito T, Moriya H, Takahashi K (2007). Glial phosphorylated p38 MAP kinase mediates pain in a rat model of lumbar disc herniation and induces motor dysfunction in a rat model of lumbar spinal canal stenosis. Spine (Phila Pa 1976).

[52] Han P, Liu S, Zhang M, Zhao J, Wang Y, Wu G, Mi W (2015). Inhibition of spinal Interlukin-33/ST2 signaling and downstream ERK and JNK pathways in electroacupuncture analgesia in formalin mice. PLoS One.

[53] Suzuki M, Inoue G, Gemba T, Watanabe T, Ito T, Koshi T, Yamauchi K, Yamashita M, Orita S, Eguchi Y (2009). Nuclear factor-kappa B decoy suppresses nerve injury and improves mechanical allodynia and thermal hyperalgesia in a rat lumbar disc herniation model. Eur Spine J.

[54] Luo JG, Zhao XL, Xu WC, Zhao XJ, Wang JN, Lin XW, Sun T, Fu ZJ (2014). Activation of spinal NF-kappaB/p65 contributes to peripheral inflammation and hyperalgesia in rat adjuvant-induced arthritis. Arthritis Rheum.

